# PROSPECTIVE EVALUATION OF INJURIES OCURRED DURING A PROFESSIONAL SOCCER CHAMPIONSHIP IN 2016 IN SÃO PAULO, BRAZIL

**DOI:** 10.1590/1413-785220172505167238

**Published:** 2017

**Authors:** GUSTAVO GONÇALVES ARLIANI, PAULO HENRIQUE SCHMIDT LARA, DIEGO COSTA ASTUR, ANDRÉ PEDRINELLI, JORGE ROBERTO PAGURA, MOISÉS COHEN

**Affiliations:** 1. Department of Orthopedics and Traumatology, Centro de Traumatologia do Esporte, Universidade Federal de São Paulo, São Paulo, SP, Brazil.; 2. Instituto de Ortopedia e Traumatologia, Hospital das Clínicas, Faculdade de Medicina da Universidade de São Paulo, São Paulo, SP, Brazil.; 3. Department of Neurology and Neurosurgery, Faculdade de Medicina do ABC, Santo André, SP, Brazil.

**Keywords:** Soccer, Athletes, Wounds and injuries, Epidemiology

## Abstract

**Objective:**

To identify the incidence of injuries, their main characteristics, and the way they were managed throughout 2016 in two major series of a professional soccer championship in São Paulo, Brazil.

**Methods:**

This prospective study used an electronic questionnaire previously developed by the Medical Committee of the Paulista Soccer Federation which was sent to the team doctors after each match.

**Results:**

Two hundred and fifty-nine injuries occurred during 361 matches, and the incidence of injury per 1000 hours of game play was 21.32. Strikers were the most affected by injury; the most frequent diagnosis was muscle injury and the legs were predominantly affected. Most of the injuries occurred in the last 15 minutes of the first half and only 7.7% required surgical treatment.

**Conclusions:**

Muscle injuries were the most frequent, with most occurring in forwards and in the legs. Approximately half of the injuries occurred after contact and the vast majority was treated without surgery. MRI was the most requested exam and most injuries were classified as moderate (8 to 28 lost play days). ***Level of Evidence III, Study of Non Consecutive Patients; Without Consistently Applied Reference “Gold” Standard.***

## RESUMO

**Objetivo:**

Identificar a incidência de lesões, suas principais características e a maneira como foram conduzidas durante todo o ano de 2016 nas duas principais séries (A1 e A2) do Campeonato Paulista.

**Métodos:**

Realizou-se um estudo prospectivo por meio de questionário eletrônico previamente desenvolvido pelo Comitê Médico da Federação Paulista de Futebol, que foi enviado aos médicos dos times das séries A1 e A2 do Campeonato Paulista de Futebol após cada rodada.

**Resultados:**

Houve 259 lesões durante 361 jogos, com incidência de 21,32 lesões por 1.000 horas de jogo ao se agrupar as duas séries do Campeonato Paulista. Os atacantes foram os mais envolvidos, sendo as lesões musculares as mais frequentes e os membros inferiores os mais afetados. A maioria das lesões ocorreu nos últimos 15 minutos do primeiro tempo e somente 7,7% das lesões precisaram de tratamento cirúrgico.

**Conclusões:**

As lesões musculares são as mais frequentes, sendo que a maioria ocorreu em atacantes e nos membros inferiores. Cerca de metade das lesões ocorreu após contato e a maioria absoluta das lesões foi tratada de forma não cirúrgica. A ressonância magnética foi o exame mais solicitado e a maior parte das lesões foi classificada como de gravidade moderada (8 a 28 dias de afastamento). **Nível de Evidência III, Estudo de Pacientes Não** ***-*** **Consecutivos; Sem Padrão de Referência “Ouro” Aplicado Uniformente.**

## INTRODUCTION

Soccer is the most popular sport in the world, with an estimated 240 million amateur athletes and at least 200,000 professional athletes. It is a sport that covers all age ranges, both sexes, and presents a high rate of injuries (70 injuries per 1000 hours of play).^1^ Soccer features short, fast, and non-continuous movements like acceleration, deceleration, changes in direction and leaps, and also involves extensive contact, which leads to injuries.^2^ ^,^ ^3^

National entities from countries as USA and UEFA tend to characterize and disclose injuries from their major championships in order to develop programs to prevent and reduce morbidity caused by soccer-related injuries. Previous studies have reported that muscle injuries, contusions, and sprains comprise 75% of injuries in professional soccer players, with the majority affecting i the legs (60-85%).^4^

Brazil has a large number of practitioners and is considered to have some of the best players in the world. The data are sparse and little is known about types of injuries and how and when they occur, which makes it difficult to prevent and treat these injuries and to rehabilitate the players.^5^

The objective of this study was to identify the incidence of injuries, their main features, and how they were handled throughout 2016 during the two main series (A1 and A2) of the Campeonato Paulista (São Paulo State Championship).

## MATERIALS AND METHODS

This research project was approved by the institutional review board (number 56723616.3.0000.5505).

This prospective study was conducted using an electronic questionnaire previously developed by the Medical Committee of the Paulista Soccer Federation and sent to the physicians for the teams in series A1 and A2 after each round of the 2016 São Paulo State Championship. All participants signed the Informed Consent Form prior to participating in the study.

A questionnaire was sent after each round to assess the incidence of injuries and their characteristics. The questionnaire consisted of 10 questions about the characteristics of the game, the athlete, and of the injury. (Annex 1)

The concept used to define soccer injuries was the same chosen by Fuller et al.^6^ for the 2005 FIFA consensus, describing them as: “Any physical complaint sustained by a player that results from a football match or football training, irrespective of the need for medical attention or time loss from football activities.”

To evaluate the outcome of the injuries reported, a questionnaire was sent for each injury which occurred and was completed after the athlete returned to training and game play. The questionnaire was comprised of six questions spanning from the complementary examination performed to the final diagnosis. (Annex 2)

To obtain the game schedules, we requested the game records from the Paulista Soccer Federation and divided the schedules as follows: morning (start before noon), afternoon (start before 6 p.m.), and night (start after 6 p.m.).

To assess the risk of injury we calculated the incidence of injury, which is expressed by the number of injuries per 1000 hours of exposure.^6^ ^,^ ^7^ To calculate exposure in games we used the following formula:

Exposure = number of injuries in games x number of players participating in the game x game duration in minutes / 60’

To calculate the incidence in games we used the following formula:

Incidence = number of injuries in games x 1000 hours/time of exposure 

### Statistical analysis

We used statistical tests because the data were quantitative and continuous. We used the equality of two proportions test to characterize the distribution of the relative frequency of the qualitative variables. Differences with p<0.05 were considered statistically significant. SPSS V17 software was used to conduct the analysis.

## RESULTS

The mean age of the injured players was 26.8 years, and the mean number of days lost as a result of injuries was 23.2. The fewest games took place in the morning (11.2%), 34.1% of the games took place in the afternoon, and 54.7% took place at night.

**Figure 1:**
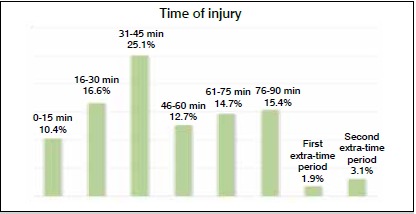
Time of injury.

During a total of 361 games 259 injuries were described, with an average of 0.71 injuries per game. Of the total, 27% of the injuries occurred in strikers, 17.4% in attacking midfielders, 17.4% in defensive midfielders, 17% in full-backs, 15.8% in central-backs, and 5.4% in goalkeepers. Most injuries occurred at the end of the first half, between the 31- and 45-minute mark (25.1%). (Figure 1)

**Figure 2:**
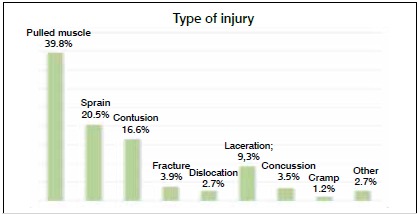
Type of injury.

As for location, the most frequent injuries were to the legs (73.4%), head (15.1%), arms (6.2%), and trunk (5.4%). The right side was most frequently affected (45.6%) and 17.8% of injuries were not classifiable as one side or the other. Contact was involved in 49% of the injuries. As for type of injury, muscle strains were the most common (39.8%), followed by sprains (20.5%) and contusions (16.6%). (Figure 2) The most common initial diagnoses were hamstring muscle injury (23.9%), adductor muscle injury (7.7%), injury to the lateral ligament of the ankle (5.8%), quadriceps muscle injury (5%), concussion (3.9%), and facial cut/contusion (3.9%).

During series A1, there were 24.16 injuries per 1000 hours of play, and in series A2 there were 19.10 injuries per 1000 hours of play. For the two series combined, there were 21.32 injuries per 1000 hours of game play.

**Figure 3:**
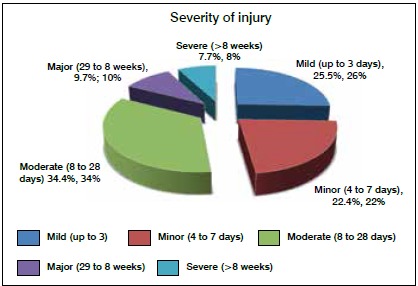
Severity of injuries.

When requested, the most common complementary exams performed were magnetic resonance imaging (36.7%) and ultrasound (28.2%), followed by X-rays (15.4%) and computed tomography (6.6%). Only 7.7% of the injuries required surgery. The most common injuries (34.4%) were considered moderate according to the severity scale, with lost time of 8 to 28 days. (Figure 3)

## DISCUSSION

The mean number of days the athletes lost per injury was 23.2. There was an incidence of 21.32 injuries per 1000 hours of game play for both series of the São Paulo State Championship soccer championship combined. Most injuries occurred during the last 15 minutes of the first half and only 7.7% of the injuries required surgical treatment.

A number of studies have investigated the incidence and main causes of soccer injuries.^1,7-11^ The mean age of the injured players in this present study was 26.8 years, higher than described in the literature.^5^ ^,^ ^12^ On average, athletes lost 23.2 days of play after injury, more than indicated in the study by Stubbe et al.,^13^ who found an average of 8 days of lost play. One possible explanation for this high average was the presence of 10 cases of tears in the anterior cruciate ligament of the knee, which led to an average of more than 6 months of lost play. We found an average of 0.71 injuries per game, below the number found in several studies in the literature including Pedrinelli et al.^1^ and Junge and Dvorak,^14^ who found 2.4 injuries per game. This difference may be because our study assessed more players than the other studies.

As for prevalence according to the diagnosis, contusions, muscle strains and sprains are the most frequent injuries in the literature, most commonly in the legs.^1^ ^,^ ^15^ ^-^ ^18^ Our study shows similar results; not only were leg injuries the absolute majority (73.4%), but muscle strains and sprains were most common. Strikers were the most affected by injuries; this finding counters the results of previous studies in which midfielders were most affected.^19^ ^,^ ^20^ This may be because these studies did not divide midfield players into defensive and attacking midfielders as we did in this present study. As for the incidence of injuries per 1000 hours of play, we found results that are within the range found in the literature, from 15 to 70 injuries per 1000 hours of game play.^1,21-23^ It should be noted that the values vary so widely in previous studies because of differences in study design, data collection methods, and the definition of injury.^24^ Our study also differed from the literature in terms of the time of injury, which was more common between the 31 and 45 minute mark, while other studies have shown that most injuries occurred in the last 30 minutes of the game.^1,22^ In our study, 49% of the injuries occurred after contact, results which are similar to the findings by Pedrinelli et al*.*,^1^ but less than the literature in which more than 70% of injuries occur after contact.^22^ ^,^ ^23^

The most commonly requested supplementary examination after injury was magnetic resonance imaging; this may result from the fact that muscle injuries were most common, and these types of injuries are generally assessed via this examination. In our study, most injuries were considered moderate (8 to 28 days of lost play), which is similar to the findings by Stubbe et al.^13^ but differs from Pedrinelli et al.^1^ and Cohen et al.,^12^ who found mild injuries (4 to 7 days lost) to be most common. Only 7.7% of the injuries required surgery, which results from the fact that the vast majority of injuries which affect soccer players (such as muscle strains and contusions) are managed conservatively.

This study has some methodological limitations. There is the possibility of outcome information bias, since precise data on the injuries may have been altered or even omitted by the team physicians. Furthermore, the study evaluated acute injuries which occurred during games, and consequently chronic injuries as well as those which occurred during practice and diseases unrelated to sports were not recorded. In a study conducted during the 2010 World Cup by Dvorak et al.,^25^ injuries that occurred during practice had very different diagnoses than those which occurred during the game play, but the severity of the injuries was similar and non-sports diseases affected approximately 12% of the players. We believe it is important to expand medical supervision for injuries during practice and off-field diseases in the players. Another point was that exposure time was calculated using 22 players and 90 minutes of play. A more precise method would be to consider extra time or the real time for each game and the number of minutes of exposure for each individual player.

The information in this study is critical to preventing new injuries in soccer. The data will allow athletes and medical staff in clubs and federations to carry out preventative programs aiming at reducing the incidence of injuries in the sport.

## CONCLUSIONS

Muscle injuries were the most frequent, with most injuries affecting strikers and the legs. Approximately half of the injuries occurred after contact and most were treated non-surgically. Magnetic resonance imaging was the most frequently requested exam and most of the injuries were classified as moderate (with 8 to 28 days lost). 
